# Ichthyofauna Used in Traditional Medicine in Brazil

**DOI:** 10.1155/2012/474716

**Published:** 2012-02-01

**Authors:** Ana Carla Asfora El-Deir, Carolina Alves Collier, Miguel Santana de Almeida Neto, Karina Maria de Souza Silva, Iamara da Silva Policarpo, Thiago Antonio S. Araújo, Rômulo Romeu Nóbrega Alves, Ulysses Paulino de Albuquerque, Geraldo Jorge Barbosa de Moura

**Affiliations:** ^1^Laboratory of Fish Ecology, Department of Biology, Federal Rural University of Pernambuco, 52171-900 Recife, PE, Brazil; ^2^Ethnozoology, Conservation and Biodiversity Research Group, Department of Biology, State University of Paraíba, 581097-53 Campina Grande, Brazil; ^3^Laboratory of Applied Ethnobotany, Department of Biology, Federal Rural University of Pernambuco, 52171-900 Recife, PE, Brazil; ^4^Laboratory of Herpetology and Paleoherpetology, Department of Biology, Federal Rural University of Pernambuco, 52171-900 Recife, PE, Brazil

## Abstract

Fish represent the group of vertebrates with the largest number of species and the largest geographic distribution; they are also used in different ways by modern civilizations. The goal of this study was to compile the current knowledge on the use of ichthyofauna in zootherapeutic practices in Brazil, including ecological and conservational commentary on the species recorded. We recorded a total of 85 species (44 fresh-water species and 41 salt-water species) used for medicinal purposes in Brazil. The three most commonly cited species were *Hoplias malabaricus, Hippocampus reidi,* and *Electrophorus electricus*. In terms of conservation status, 65% of species are in the “not evaluated” category, and 14% are in the “insufficient data” category. Three species are in the “vulnerable” category: *Atlantoraja cyclophora*, *Balistes vetula,* and *Hippocampus erectus*. Currently, we cannot avoid considering human pressure on the population dynamics of these species, which is an essential variable for the conservation of the species and the ecosystems in which they live and for the perpetuation of traditional medical practices.

## 1. Introduction

Nature offers various resources that people use to guarantee their survival [1] and to reproduce their ways of life and their practices. The use and management of these resources is intimately linked with the needs of various human populations. Among traditional populations, the use of plant and/or animal resources for medicinal purposes has been reported by various authors as an essential practice in traditional medical systems [2–13]. Natural resources have been used in traditional medical practices since ancient times, and their use is spreading in contemporary society [14]. One very old alternative therapy involves the use of animals and their derivatives in the production of zootherapeutic medications [15]. Zootherapy is an important alternative for cures in local populations; it can also be useful for the development of new drugs in modern medicine [4].

In Brazil, zootherapy appears well established; its broad biological diversity, along with its cultural complexity, drive production of zootherapeutic products [16]. In addition, the difficulty in accessing the main health system encountered by some populations increases the demand for traditional medicine [17].

Among the animal taxa used as medicinal resources, fish deserve special attention due to their strong representation in zootherapeutic surveys in Brazil [2, 7, 8, 12, 30, 42]. As a resource, fish are exploited in different ways by each culture [36]. Their medicinal applications include the use of both body parts and materials produced by the fish, along with live individuals [22].

Many of the animals used medicinally are found on the list of endangered species [7]; the risk of extinction is not only for the species but also for the benefits they offer. One of the benefits resulting from research in zootherapy is the discovery of new compounds that have pharmacological potential [21]. Given what has been stated above, this study aims to gather the current knowledge on ichthyofauna used in zootherapeutic practices in Brazil. By doing so, we expect to broaden the knowledge base through a compilation of species used to provide a first approximation of the wealth of these resources and their potential. Additionally, the study will evaluate whether the habitat of these species influences its versatility of use and if there are differences in the diversity of species cited for each body system.

The information compilation was based on bibliographic data. We considered bibliographic data from book chapters, in periodicals publications, and technical information available in online databases. We only considered a valid taxa the identified on species level, since the use of clades identified on the genus level, without its proper description, does not allow the technical-scientific accumulation of the taxon, which justifies this compilation with a fewer species number when compared to Costa-Neto and Alves [42] and R. R. N. Alves and H. N. Alves [13].

The database generated contains information on taxonomy, habitat, conservation status through the IUCN, the part of the animal used, therapeutic indications, and the Brazilian states where the species were cited. Species nomenclature, their habitats, and conservation status were confirmed and updated according to [43–45].

Though the locations sampled employed different methods and collection efforts, we counted the numbers of species used for zootherapeutic purposes by Brazilian region (state) and therapeutic indication. While it was not possible to perform a refined comparative analysis on the distribution of species use, this method allowed us to record the breadth of geographic distribution of the zootherapeutic indications and the study frequency by Brazilian regions and states.

We used the Index of Relative Importance (IR) [46] to measure the versatility of use of each species. This index takes into consideration the properties attributed and the body systems that are indicated for each species. This index varies from 0 to 2, with 2 indicating the most versatile species. We used the Kruskal-Wallis test to evaluate whether the relative importance of a species was related to its habitat (i.e., salt water or fresh water) and its conservation status. We also compared habitats relative to species wealth for each body system using the Kolmogorov-Smirnov test. BioEstat v.5.0 software was used for analysis [47].

Therapeutic indications were categorized according to body systems from [48]: digestive, respiratory, gynecological/urinary, circulatory, nervous, sensory, motor, puerperal, cutaneous, scarring, poisoning, neoplasia, hematopoietic, nutrition, infectious/parasitic, lack of sexual desire, anti-abortive, and postpartum. Indications that could not be classified in these systems were grouped as “undefined pains/disorders.” 

## 2. Ichthyofauna in Traditional Medical Practices in Brazil

**Figure 1:**
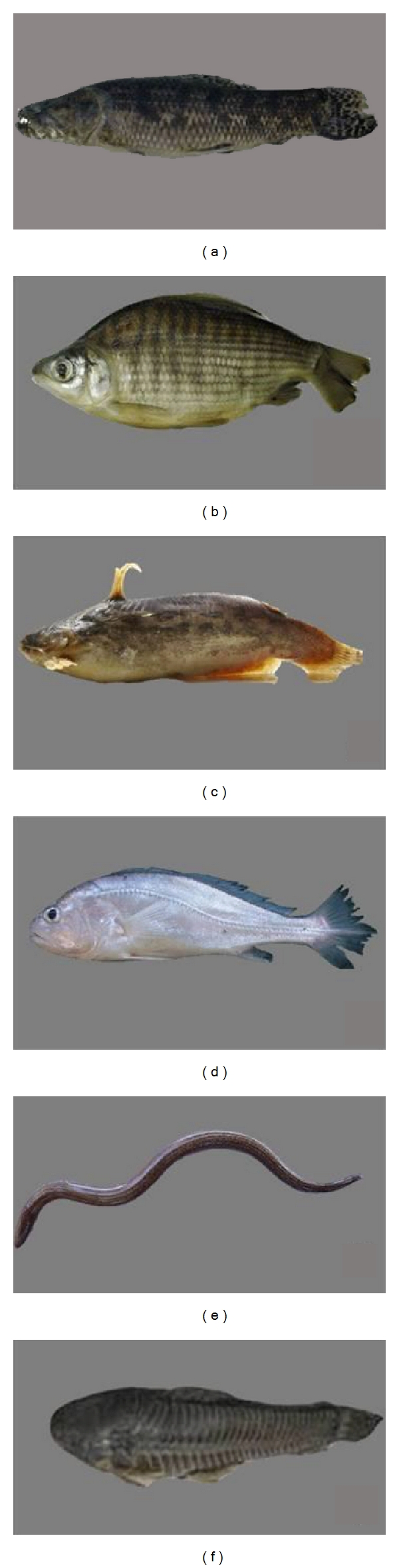
Species cited in the traditional medicine of Brazil ((a) *Hoplias malabaricus *(Bloch 1794) (Trahira/Traíra), (b) *Prochilodus argenteus *Spix and Agassiz, 1829 (curimatã), (c) *Trachelyopterus galeatus *(Linnaeus, 1766) (Driftwood catfishes/Cumbá), (d) *Plagioscion squamosissimus *(Heckel, 1840) (South American silver croaker/Corvina), (e) *Synbranchus marmoratus *Bloch, 1795 (marbled swamp eel/muçum), (f) *Callichthys callichthys *(Linnaeus, 1758) (cascudo/caboje)).

The inventory of ichthyofauna used in Brazilian zootherapy produced a list of 85 species, of which 44 are predominantly fresh water and 41 are predominantly salt water fishes; 22 are cartilaginous fish (Figure 1). The most commonly listed fish were Hoplias malabaricus (Bloch, 1794) (*N* = 15), followed by Hippocampus reidi Ginsburg, 1933 (*N* = 13), and Electrophorus electricus (Linnaeus, 1766) (*N* = 10) (Table 1). These three species are highly important for zootherapy due to their documented use in various regions of Brazil [24–26, 31, 36]. 

These most frequently used fish resources are part of the native fauna, demonstrating the importance of local fauna as a source for traditional remedies. According to R. Alves and H. Alves [13], the composition and availability of fauna are factors that directly affect the composition of the local zootherapeutic arsenal.

The dissemination of zootherapeutic knowledge is reflected in the population's contact with resources that, in principle, are not available locally. Some species that are restricted to the coast, such as the seahorse (*Hippocampus reidi*), are broadly disseminated throughout the interior of Brazil [24, 25, 27]. The use of this species was recorded for populations in the interior, such as the cities of Santa Cruz do Capibaribe-PE [24], Crato-CE [33], Queimadas-PB [25], and Caruaru-PE [26]. This situation may be explained by the existence of commercial routes for medicinal animals involving different cities in Brazil [49]. An exotic species such as the cod *Gadus morhua *Linnaeus, 1958, is available commercially in various states in Brazil for culinary purposes, but it is also used medicinally in states such as Paraíba and Bahia [2, 23].

**Figure 2:**
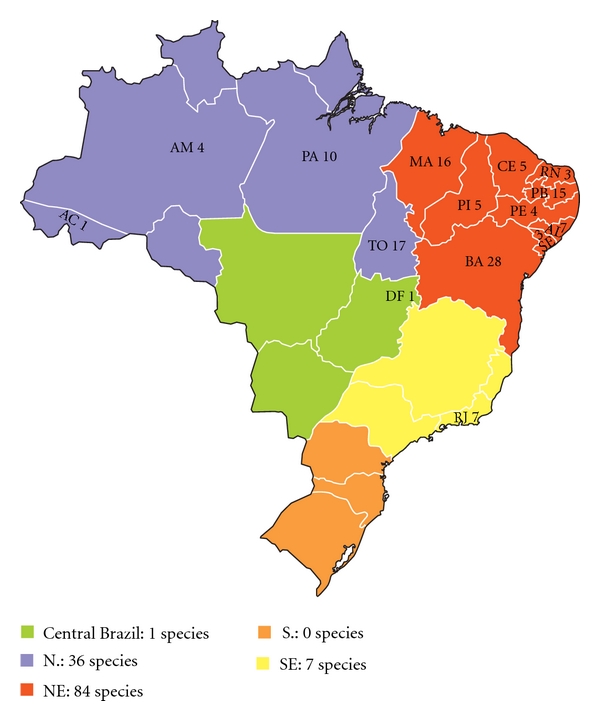
Distribution of richness of ichthyofauna used in traditional medicine in Brazil.

Zootherapeutic practice involving ichthyofauna was recorded in 14 Brazilian states, representing the North, Northeast, Center-West, and Southeast regions. The state of Bahia (28 spp.) had the highest number of fishes used as traditional remedies, followed by the states of Tocantins (21 spp.), Paraíba (19 spp.), Maranhão (16 spp.), and Pará (9 spp.). This may not reflect the true situation regarding zootherapy in Brazil; the number is likely underestimated due to the concentration of studies in these regions (Figure 2).

The Northeast region was the best represented, with research performed in eight states: Piauí, Maranhão, Ceará, Rio Grande do Norte, Paraíba, Pernambuco, Alagoas, and Bahia. This region has a significant presence of zootherapy in curing practices [7, 50]. Alves [12], while recording zootherapeutic practices in this region, did not perform studies in Ceará and Rio Grande do Norte; however, studies performed that same year [27, 33] and in the following year [28] demonstrated the medicinal use of animals in these two states. The North region was the second-most frequently represented, followed by the Southeast and Center-West regions, which accounted for 7% of the studies.

## 3. Therapeutic Indications for Ichthyofauna

Various therapeutic indications have been associated with ichthyofauna for medicinal use in Brazil, with 83 different diseases or illnesses recorded, particularly asthma, rheumatism, wounds, alcoholism, and bronchitis.

*Hippocampus reidi *and *Hippocampus erectus *stand out among the salt water species, with RI (relative importance) values of 1.73 and 0.98, respectively. The importance of these species is also evident from the number of studies that reported them in their inventories, especially in Northeast Brazil.

*Hoplias malabaricus *scored highest on diversity among the predominantly fresh water species, with an RI of 2.00, the highest score among all the species in the inventory. This species also stood out regarding the number of parts of the fish that can be used in traditional remedies. *Electrophorus electricus *received the second-highest RI score (1.60). It was also evident that these species have regional importance, due to the fact that they are cited in various studies conducted in Northern and Northeast Brazil. There was no significant difference between the species regarding habitat, according to the Kruskal-Wallis test (*H* = 1.213; *P* = 0.270).

**Figure 3:**
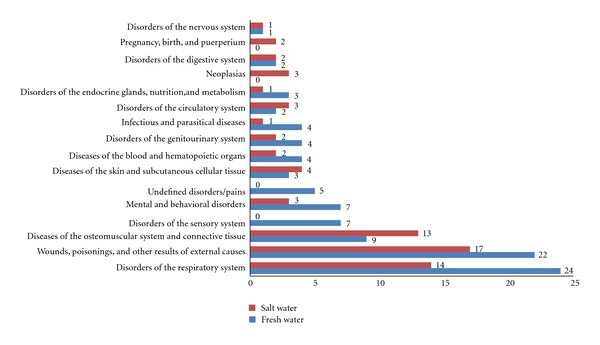
Body systems by fish species used in zootherapeutic practices in Brazil.

The therapeutic indications were grouped into 16 body systems (Figure 3). Of these, only two categories did not appear for the fresh water species: neoplasias and problems relating to pregnancy, birth, and puerperium. Two categories did not appear among salt water species: sensory system disorders and undefined pains/disorders.

The systems with the greatest diversity of species included disorders of the respiratory system (e.g., asthma, bronchitis, and pneumonia) and wounds, poisonings and other results from external causes (e.g., wounds caused by the fish itself, burns, and scarring). In spite of the fact that 57% of systems had greater diversity for fresh water than for salt water species, no significant differences in species wealth were observed (*P* = 0.374) between the two groups. 

Often, a single species is the source of treatment for many diseases and infirmities [27]. Among the most versatile species are *Hoplias malabaricus, Electrophorus electricus, Hippocampus reidi, Hippocampus erectus, and Phractocephalus hemiliopterus*. The trahira (*Hoplias malabaricus*) was very versatile in treating 35% of therapeutic indications, ranging from bone and respiratory problems to alcoholism and snakebite. The electric eel (*Electrophorus electricus*) and the longsnout seahorse (*Hippocampus reidi*) treated 23% of indications each, and the redtail catfish (*Phractocephalus hemiliopterus*) and another species of seahorse (*Hippocampus erectus*) each treated 12%. It should be noted that seahorses and the trahira are heavily commercialized in Northeast Brazil [9, 51].

Although a particular species can be associated with various indications, these therapeutic uses may be associated with the use of different parts of the animal. The head of *Hoplias malabaricus *(trahira) is used for treatment of tetanus [38], while its scales are used to combat stroke [20], and the fat and skin secretion are indicated as a remedy for alcoholism [16, 27]. Another example of therapeutic versatility is found in *Electrophorus electricus *(electric eel), whose bones are used to treat snakebite [31], while the fat is associated with other indications, such as pains [26, 28, 31], rheumatism [7–9, 17, 26, 27, 31, 37], colds [31], asthma [31, 37], and pneumonia [8, 37]. 

Among the fish parts most commonly employed for the production of zootherapeutic products, fat stood out with a 40% use occurrence. Fish fat is indicated for various infirmities and diseases. Its use recurs often in popular medicine [31]; fat has been documented as the most commonly used animal part in various studies [8, 35]. In India, the fat from various animals is indicated for combating all types of pain, impotence, burns, and paralysis [52]. The widespread use of fat can be related to the ease of its extraction. Additionally, it can be preserved at room temperature for long periods [29].

**Figure 4:**
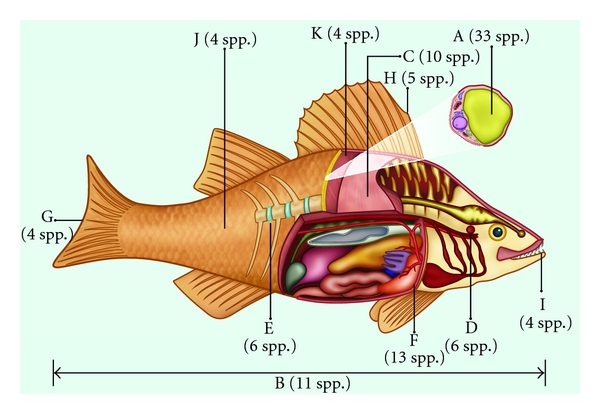
Richness of species according to the main body parts of fish used for therapeutic purposes in Brazil. (A: fat, B: entire, C: meat, D: otoliths, E: cartilage, F: liver, G: tail, H: spur, I: tooth, J: scale, K: skin).

The use of various other parts of fish has also been recorded, including teeth, eyes, gall, liver, wattles, otoliths, fins, and stingers. Many fish parts used in zootherapy are not used for other purposes, such as scales and leathers, to maximize the use of local resources [35]. Another method for keeping therapeutic resources available are food taboos, through which the consumption of some of these species would lead to negative consequences, thereby keeping these animals available in case of necessity (Figure 4) [31].

In addition to dead animals and their parts, the use of living animals is a recurring practice in traditional medicine systems and is a part of the beliefs and “spells” in local systems [22]. A mystical use has been reported for the species *Synbranchus marmoratus *(marbled swamp eel) and *Callichthys callichthys *(armored catfish) [39] in the treatment of asthma; namely, one should spit in the mouth of a living animal, and then put it back in the river.

Another demonstration of aspects associated with popular medicine occurs when the morphology exhibited by the animal inspires its therapeutic application. Sometimes the morphology of the animal and/or the organs utilized is associated with the part of the human body to be treated because the similarities are interpreted as indicative of a potential benefit [35]. Moura and Marques [35] recorded the use of the common wood catfish (*Trachelyopterus galeatus*) in the treatment of impotence, due to the species' large, fringed testicles.

Zootherapy has been the focus of increasing attention from the pharmaceutical industry [7]. These industries have used the biologically active components present in traditional medicines as sources for the production of many drugs [53]. Compounds extracted from fish are already used in official medicine, such as Tetrodotoxin, which originates from pufferfish and possesses a powerful anesthetic effect [54–56]. Other widely distributed compounds from fish, omega-3 fatty acids, are associated with the prevention and treatment of cardiovascular diseases, arthritis, kidney disease, and inflammation [57].

**Figure 5:**
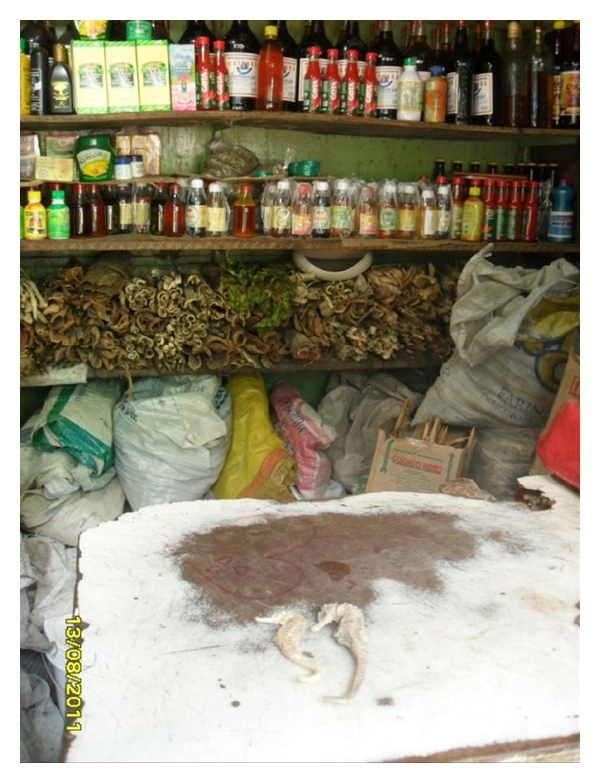
Box in the Market of São José (Recife, Brazil) with seahorse to sell.

The exploitation of medicinal fauna resources by local populations and the pharmaceutical industry has had a negative impact on several species, with their survival threatened by overexploitation [10, 58]. Among the fish used therapeutically in Brazil, three species can be singled out as having an elevated danger of extinction and are included in the “vulnerable” category by the IUCN [59]: *Atlantoraja cyclophora*, *Balistes vetula, *and *Hippocampus erectus*. *Sphyrna lewini *is in the “in danger” category, with a very high risk of extinction, and *Pristis perotetti *and *Pristis pectinata *are “in critical danger.” Among these species are four cartilaginous fishes that have low levels of fecundity, such as the ray, the hammerhead shark, and the swordfish. Seahorses (*Hippocampus *spp.) are considered susceptible to exploitation and are threatened worldwide due to excessive use and destruction of habitat due to their high monetary value and potential for commercialization [51] (Figure 5). The species *H. reidi*, currently listed in the “insufficient data” category, is widely commercialized for medicinal purposes throughout Brazil and exhibits low reproduction and high mortality rates in initial phases [60]. 

**Figure 6:**
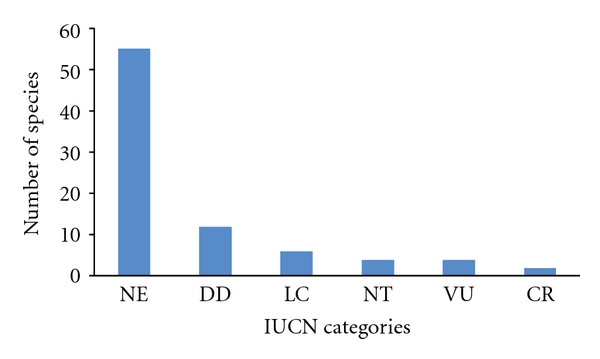
IUCN categories of fish species used for medicinal purposes in Brazil **(**EN: endangered, VU: vulnerable, LC: least concern, CR: critically endangered, NT: near threatened, NA: not available, DD: insufficient data).

However, the great majority of fish identified in this survey have not yet been evaluated by the IUCN, or there is insufficient data for analysis (Figure 6). This fact highlights the scarcity of knowledge regarding the true situation of these fish, demonstrating the need for studies directed toward those species that are used medicinally to preserve these resources and all aspects linked to them. Also, there is no significant differences in the relative importance (RI) between IUCN categories according to the Kruskall-Wallis test (*P* > 0.05).

In addition, the extinction of some species could compromise both traditional knowledge and the discovery of new drugs [61] because these species could disappear before science becomes aware of their potential. The growing demand for the biotic resources used in traditional medicine is due to the increasing quantity of studies that demonstrate the efficacy of their use, drawing the attention of the pharmaceutical industry [62]. 

Extractivism is generally the only method for obtaining zootherapeutic resources, highlighting the need to add these species to conservation efforts by including creatures involved in zootherapeutic practices in planning for the management of fauna. Both the local population and the pharmaceutical industries can contribute in different ways to the maintenance of these resources. In addition, it is also necessary to understand the ecology and biology of the species used in medicine to propose effective strategies for managing these resources.

## Figures and Tables

**Table 1:** Fish speciesused in traditional medicine in Brazil with local name, IUCN categories (EN: endangered, VU: vulnerable, LC: least concern, CR: critically endangered, NT: near threatened, NA: not available, DD: insufficient data), part used, therapeutic indication, number of bodily systems, state (occurrence), RI (relative importance), and reference.

Taxa/local name	IUCN	Part used	Therapeutic indication	Number of bodily systems	State	RI	Reference
Predominantly salt water							

**Chondrichthyes**							
**Orectolobiformes**							

Ginglymostomatidae							

*Ginglymostoma cirratum * (Bonnaterre, 1788)	DD	Cartilage	Rheumatism	1	MA, PB	0.138	[7, 9]
(Nurse shark/Cação-lixa)							
**Carcharhiniformes**							

Carcharhinidae							

*Carcharhinus limbatus * (Müller and Henle, 1839)	NT	Cartilage, fat	Osteoporosis	1	MA	0.138	[7–9, 16]
(Blacktip shark/Sucuri-da-galha-preta)							
*Carcharhinus porosus * (Ranzani, 1839)	DD	Cartilage, fat	Asthma, rheumatism, wounds, inflammations, osteoporosis	3	MA, BA, PA	0.492	[7, 9]
(Smalltail/Cação-do-salgado)							
*Carcharhinus leucas * (Muller and Henle, 1839) (Bull shark/Tubarão)	NT	—	—	—	PE	—	[3]
*Galeocerdo cuvier * (Péron and Lesueur, 1822)	NT	Cartilage, fat	Osteoporosis	1	MA, PI	0.138	[7, 9]
(Tiger shark/Jaguará)							
*Rhizoprionodon lalandii * (Müller and Henle, 1839)	DD	Cartilage, fat	Rheumatism	1	PB, BA	0.138	[7, 9, 16]
(Sharpnose shark/cação-frango) *Rhizoprionodon porosus * (Poey, 1861) (Caribbean sharpnose shark/Cação de praia)	LC	Cartilage, fat	Rheumatism	1	PB, BA	0.138	[7, 9, 16]

Sphyrnidae							

*Sphyrna lewini * (Griffith and Smith, 1834)	EN	Liver oil	Asthma, wounds, rheumatism	3	BA	0.415	[2]
(Scalloped hammerhead/Cação-martelo)							
**Pristiformes**							

Pristidae							

*Pristis perotteti * Muller and Henlle 1842	CR	Rostral expansion	Rheumatism, arthritis	1	PA	0.138	[9]
(Sawfish/Espadarte)							
*Pristis pectinata * (Latham, 1794) Smalltooth sawfish/espadarte	CR	Rostral expansion	Asthma, rheumatism, arthritis	2	—	0.135	[18]
**Rajiformes**							

Narcinidae							

*Narcine Braziliensis * (Olfers, 1831)	DD	Fat	Tooth pain	1	BA	0.138	[2, 4]
(Brazilian electric ray/Raia elétrica)							
Rajidae							

*Atlantoraja cyclophora * (Regan, 1903)	VU	Eggs	Postpartum hemorrhage	1	RJ	0.138	[19]
(Eyespot skate/Almofadinha/barata do mar)							

Dasyatidae							

*Dasyatis guttata * (Bloch and Schneider, 1801)	DD	Teeth, liver oil, tail, ventral mucus, liver	Asthma, wounds caused by the fish itself, burns on the skin	2	PB	0.315	[7, 9]
(Longnose stingray/Raia branca)							
*Dasyatis marianae * Gomes, Rosa, and Gadig, 2000	DD	Teeth, liver oil, tail, ventral mucus, liver	Asthma, wounds caused by the fish itself, burns on the skin	2	PB	0.315	[7, 9]
(Brazilian large-eyed stingray/raia mariquita)							

Myliobatidae							

*Aetobatus narinari * (Euphrasen, 1790)	NT	Teeth, liver oil, tail, ventral mucus, liver	Asthma, wounds caused by the fish itself, burns on the skin, and hemorrhages	3	PA, PI, PB	0.453	[7, 9]
(Spotted eagle ray/raia-chita)							

Urotrygonidae							

*Urotrygon microphthalmum * (Delsman, 1941)	LC	Teeth, liver oil, tail, ventral mucus, liver	Asthma, wounds caused by the fish itself, burns on the skin	2	PB	0.315	[7, 9]
(small-eyed, round stingray/raia)							
**Actinopterygii**							
**Elopiformes**							

Megalopidae							

*Megalops atlanticus * Valenciennes, 1847	NE	Scale	Asthma, lack of air, headache, stroke	3	MA, PB, AL	0.454	[7–9, 20]
(Tarpon/Camurupim/Cangurupim)							
**Anguilliformes**							

Muraenidae							

*Gymnothorax funebris * Ranzani, 1840	NE	Meat	Wounds	1	PB	0.138	[7, 9]
(Green moray/moréia verde)							
*Gymnothorax moringa* (Cuvier, 1829)	NE	Meat	Wounds	1	PB	0.138	[7, 9]
(Spotted moray/moréia pintada)							
*Gymnothorax vicinus * (Castelnau, 1855) (Purple mouth moray/moréia)	NE	Meat	Wounds	1	PB	0.138	[7, 9]

**Clupeiformes**							

Clupeidae							

*Opisthonema oglinum * (Lesueur, 1818) (Atlantic thread herring/sardinha)	NE	Entire	Alcoholism	1	PB	0.138	[7, 9]
**Siluriformes**							
Ariidae							

*Bagre bagre * (Linnaeus, 1758)	NE	Entire	Wounds caused by the fish itself	1	BA	0.138	[2, 21]
(Coco sea catfish/bagre-fidalgo)							
*Genidens barbus * (Lacepède, 1803)	NE	Entire	Wounds caused by the fish itself	1	BA	0.138	[2, 21]
(White sea catfish/bagre-do-mar)							
*Genidens genidens * (Valenciennes, 1840)	LC	Eye	Wounds caused by the fish itself	1	BA	0.138	[22]
(Guri sea catfish/Bagre)							
*Aspistor luniscutis * (Valenciennes, 1840)	NE	Entire	Wounds caused by the fish itself	1	BA	0.138	[2, 21]
(bagre-urutu)							
**Gadiformes**							

Gadidae							

*Gadus morhua * Linnaeus, 1958	VU	Fat, skin	Rheumatism, furuncle, back pain	2	PB, BA	0.315	[2, 23]

(Atlantic Cod, bacalhau)
**Batrachoidiformes**							

Batrachoididae							

*Thalassophryne nattereri * (Steindachner, 1876)	NE	Meat, eye, and brain	Wounds caused by the fish itself	1	MA, PI, BA	0.138	[2, 7, 9]
(niquim)							
**Lophiiformes**							

Ogcocephalidae							

*Ogcocephalus vespertilio * (Linnaeus, 1758)	NE	Entire	Asthma, bronchitis, rheumatism, arthritis	2	MA, PB, RJ	0.354	[7–9, 19]
(Seadevil/Peixe morcego)							
**Beryciformes**							

Holocentridae							

*Holocentrus adscensionis * (Osbeck, 1765)	NE	Sting	Wounds	1	RJ	0.138	[19]
(Squirrelfish/Jaguariçá)							
**Gasterosteiformes**							

Syngnathidae							

*Hippocampus erectus * Perry, 1810 (Lined seahorse/Cavalo-marinho)	VU	Entire	Alcoholism, thromboses, impotence, diabetes, osteoporosis, heart disease, bronchitis, cancer, asthma, and rheumatism	6	Brazil	0.985	[21]
*Hippocampus reidi * Ginsburg, 1933 (Longsnout seahorse/Cavalo-marinho)	DD	Entire	Edema, asthma, bronchitis, impotence, thromboses, hemorrhage, hemorrhage in women, postpartum disorders, gastritis, tuberculosis, epilepsy, alcoholism, increasing female fertility, osteoporosis, heart disease cancer, asthma, rheumatism, avoiding miscarriage	10	RJ, PE, RN, PB, CE, BA, MA, PI, Brazil	1.731	[1, 2, 4, 7–9, 19, 24–29]
**Perciformes**							

Centropomidae							

*Centropomus undecimalis * (Bloch, 1792)	NE	Fat	Swollen legs, edema	1	BA	0.177	[2]
(Common snook/Robalo)							

Sparidae							

*Calamus penna * (Valenciennes, 1830)	NE	Fin	Asthma	1	BA	0.138	[2]
(Sheepshead porgy/peixe-pena)							

Sciaenidae							

*Cynoscion acoupa * (Lacepède 1802)	LC	Otoliths	Renal insufficiency	1	MA	0.138	[8]
(Acoupa weakfish/Pescada amarela)							
*Cynoscion leiarchus * (Curvier 1830)	NE	Otoliths, Head	Renal insufficiency, lack of air	1	MA, PB	0.177	[8, 30]
(Smooth weakfish/Pescada branca)							
*Micropogonias furnieri * (Desmarest, 1823)	NE	Otoliths	Bronchitis	1	RJ	0.138	[29]

Trichiuridae							

*Trichiurus lepturus * (Linnaeus, 1758) (largehead hairtail/peixe espada)	NE	Tail	Asthma	1	—	0.138	[18]
**Tetraodontiformes**							

Balistidae							

*Balistes vetula * Linnaeus, 1758	VU	Skin	Asthma, back pain	2	MA	0.277	[8]

(Queen-triggerfish/cangulo)
*Balistes capriscus * Gmelin, 1789	NE	Skin	Bronchitis		RJ	0.138	[29]
(Grey-triggerfish/capucho)							

Tetraodontidae							

*Colomesus psittacus * (Bloch and Schneider, 1801) (Banded puffer/Baiacú)	NE	Liver oil, bile	Breast cancer, back pain, warts	3	MA	0.415	[7, 9]

*Sphoeroides testudineus * (Linnaeus, 1758) (Checkered puffer/Baiacú)	NE	Fat	Rheumatism	1	BA	0.138	[1]

Predominantly fresh water							

**Chondrichthyes**							
**Rajiformes**							

Potamotrygonidae							

*Paratrygon ajereba * (Walbaum, 1792)	DD	Spur, Fat	Asthma, cold, cough, ear pain, pneumonia, umbilical hernia, burns on the skin	3	TO	0.569	[31]
(Discus ray/raia)							
*Plesiotrygon iwamae * (Rosa, Castello and Thorson, 1987) (Long-tailed river stingray/Arraia)	DD	Fat	Wounds caused by the fish itself, cracks on the soles of feet, wounds	1	PA	0.138	[9]
*Potamotrygon hystrix * (Müller and Henle, 1841) (Porcupine river stingray/Raia)	DD	Spur, Fat	Asthma, cold, cough, ear pain, pneumonia, umbilical hernia, burns on the skin	3	TO	0.569	[31]
*Potamotrygon motoro * (Müller and Henle, 1841) (South American freshwater stingray/Raia)	DD	Spur, Fat	Asthma, cold, cough, ear pain, pneumonia, umbilical hernia, burns on the skin	3	TO	0.569	[31]
*Potamotrygon orbignyi * (Castelnau, 1855)	LC	Fat	Wounds caused by the fish itself	1	PA	0.138	[9]
(Smooth back river stingray/Arraia)							
**Actinopterygii**							
**Osteoglossiformes**							

Arapaimidae							

*Arapaima gigas * (Cuvier, 1829)	DD	scale	Asthma	1	PA	0.138	[8]
(Arapaima/arapaima, pirarucu)							

Osteoglossidae							

*Osteoglossum ferreirai * Kanazawa, 1966 (Black arawana/Aruanã)	LC	scale	Dermatological problems	1	AM	0.138	[32]
(Arapaima/arapaima, pirarucu)							
(Cruvina, Crumatá)		Fat, meat					
*Prochilodus nigricans * Agassiz 1829	NE	Fat, gall, meat	Inflammations, cholesterol, burns on the skin, wounds, rheumatism, chilblains, malaria, whooping cough	5	CE, TO, Brazil	0.808	[27, 33, 34]
(Black prochilodus/Curimatã, Papa-terra)							

Anostomidae							

*Leporinus piau * Fowler, 1941	NE	Fat	Rheumatism	1	BA	0.138	[35]
(Piau)							
*Leporinus steindachneri * Eigenmann 1907 (Piau)	NE	Fat	Cholesterol	1	CE	0.138	[27]

*Schizodon knerii * (Steindachner, 1875) (Piau branco)	NE	Fat	Edema, leukoma	2	AL	0.277	[20]

Characidae							

*Brycon nattereri * Günther, 1864	NE	Meat	Flu	1	BA	0.138	[36]
(Matrinchã)							
*Piaractus brachypomus * (Cuvier, 1818)	NE	Fat	Scarring	1	TO	0.138	[37]
(Pirapatinga/Caranha)							
*Serrasalmus brandtii * Lütken, 1875	NE	Tail, gall, fat	Impotency, jaundice, edema, inflammations	3	BA, AL	0.454	[20, 22, 35]
(White piranha/Piranha)							
*Mylossoma duriventre * (Cuvier, 1818) (Pacu manteiga)	NE	Fat	STDs	1	TO	0.138	[31]

Incertae sedis in Characidae							

*Astyanax cf. bimaculatus * (Linnaeus, 1758)	NE	Entire	Alcoholism	1	BA	0.138	[2]
(Two-spot astyanax/Piaba)							
*Chalceus macrolepidotus * Cuvier, 1818	NE	Entire, eye	Asthma	1	AM	0.138	[11]
(Pink tailed chalceus/Araripirá)							
*Paracheirodon axelrodi * (Schultz, 1956)	NE	Entire	Asthma	1	AM	0.138	[11]
(Cardinal tetra/Cardinal)							
*Salminus hilarii * Valenciennes, 1850	NE	Head	Memory	1	TO	0.138	[37]
(Dourado)							

Cynodontidae							

*Hydrolycus scomberoides * (Cuvier, 1819) (Payara/Cachorra)	NE	Fat	Ear pain	1	TO	0.138	[31]

Erythrinidae							

*Erythrinus erythrinus * (Bloch and Schneider, 1801)	NE	Entire	Asthma	1	AL	0.138	[20]
(Matroê)							
*Hoplias lacerdae * Miranda Ribeiro, 1908 (Trahira/Traírão)	NE	Fat	Rheumatism, “vilide”	2	BA	0.277	[35]

*Hoplias malabaricus * (Bloch 1794) (Trahira/Traíra)	NE	Fat, epidermal secretion, “bucho”, entire, head, scale, meat	Alcoholism, ear pain, inflammations, cholesterol, sore throat, umbilical cord inflammation, contusions, inflamed ear, hearing problems, ocular inflammation, urinary infection, deafness, asthma, muscle strain, erysipelas, wounds, hemorrhages, snakebite, conjunctivitis, edema, rheumatism, leukoma, stroke, asthma, diarrhea, vision problems	10	AC, BA, RN, PA, PB, MA, PE, AL, TO	2.000	[1, 2, 8, 9, 11, 16, 20, 22, 24, 27, 28, 30, 31, 33, 36, 38]
**Siluriformes**							

Cetopsidae							

*Cetopsis candiru * Spix and Agassiz, 1829	NE	Meat	Whooping cough	1	TO	0.138	[37]
(Candiru)							

Aspredinidae							

*Aspredinichthys tibicen * (Valenciennes, 1840)	NE	Barbels	Asthma	1	MA	0.138	[7, 9]
(Tenbarbed banjo/viola)							
*Aspredo aspredo * (Linnaeus, 1758)	NE	Barbels	Asthma	1	MA	0.138	[7, 9]
(Banjo/viola)							

Callichthyidae							

*Callichthys callichthys * (Linnaeus, 1758)	NE	Entire	Asthma, umbilical hernia, bronchitis, helping a child to walk earlier	3	BA, AL	0.454	[2, 20, 39]
(Cascudo/Caboje)							

Pimelodidae							

*Brachyplatystoma filamentosum * (Lichtenstein, 1819) (Kumakuma/Filhote)	NE	Fin	Cough, alcoholism	2	TO	0.277	[37]
*Phractocephalus hemioliopterus * (Bloch and Schneider, 1801) (Redtail catfish/Pirarara)	NE	Fat	Burns on the skin, rheumatism, cough, wounds, bronchitis, whooping cough, hoarseness, pneumonia, asthma, cold, umbilical hernia	3	AM, TO, Brazil	0.723	[11, 31, 34, 37]
*Pseudoplatystoma corruscans * (Spix and Agassiz, 1829)	NE	Fat	Burns on the skin	1	BA	0.138	[36]
(Spotted sorubim/Surubim)							
*Pseudoplatystoma fasciatum * (Linnaeus, 1766) (Barred sorubim/pintado)	NE	Fat, gall	Scarring, whooping cough, body pain, muscular pains, bone pain, bronchitis, stroke	5	TO	0.769	[37]
*Sorubimichthys planiceps * (Spix and Agassiz, 1829)	NE	Meat	Tuberculosis, leishmaniasis	2	TO	0.277	[31]
(Firewood catfish/Surubim-chicote)							
*Zungaro zungaro * (Humboldt, 1821) (Gilded catfish/Jaú)	NE	Fat, skin, meat	Bronchitis, asthma, burns on the skin, rheumatism, cold, ear pain, tooth pain, chilblains	6	TO	0.908	[31, 37]

Doradidae							

*Lithodoras dorsalis * (Valenciennes, 1840)	NE	Fat	Swelling	1	PA	0.138	[9]
(Rock-bacu/bacu)							
*Oxydoras niger * (Valenciennes, 1821) (Ripsaw catfish/Abotoado)	NE	Fat	Asthma, bronchitis, grippe, scarring, dry skin	3	TO	0.492	[37]

Auchenipteridae							

*Trachelyopterus galeatus * (Linnaeus, 1766) (Driftwood catfishes/Cumbá)	NE	Entire, spur	Impotence, umbilical hernia, asthma	3	BA, AL	0.415	[20, 35, 40, 41]

*Megalodoras uranoscopus * (Eigenmann and Eigenmann, 1888) (cuiú-cuiú)	NE	Fat	Rheumatism	1	TO	0.138	[31]
*Pterodoras granulosus * (Valenciennes, 1821) (Granulated catfish/cuiú-cuiú)	NE	Fat	Rheumatism	1	TO	0.138	[31]
**Gymnotiformes**							

Gymnotidae							

*Electrophorus electricus * (Linnaeus 1756) (Electric eel/Peixe Elétrico, Poraquê)	LC	Entire, fat, spin, and bone	Acne, alcoholism, asthma, itching, contusions, headache, back pain, muscular pains, wounds, swelling, spots on the skin, osteoporosis, snake bite, insect bite, pneumonia, cold, rheumatism, deafness, muscle strain, thrombosis, tuberculosis	8	RN, PE, DF, AC, PA, MA, PI, PB, TO, BA	1.608	[1, 7–9, 12, 17, 27, 28, 31, 37]
**Synbranchiformes**							

Synbranchidae							

*Synbranchus marmoratus * Bloch, 1795 (Marbled swamp eel/muçum)	NE	Entire	Making the child walk sooner, bronchitis, asthma, bronchitis, umbilical hernia	3	BA	0.454	[2, 39]
**Perciformes**							

Sciaenidae							

*Pachyurus francisci * (Cuvier, 1830) (San Francisco croaker/Cruvina, curvina-de-bico)	NE	Otoliths	Asthma, back pain, diuretic effect, renal insufficiency	3	BA	0.454	[36]
*Plagioscion squamosissimus * (Heckel, 1840)	NE	Otoliths	Kidney stones, renal insufficiency, urinary infection, hemorrhages, snake bite	3	TO	0.492	[31, 37]
(South American silver croaker/Corvina)							
*Plagioscion surinamensis * (Bleeker, 1873)	NE	Otoliths	Urinary infection, hemorrhages, snakebite	3	TO	0.41538	[31]
(Pacora/Corvina)							
